# Salicylate of Soda in Rheumatism

**Published:** 1876-11

**Authors:** A. Clark

**Affiliations:** Prof. of Pathology and Practical Medicine, College of Physicians and Surgeons, N. Y.


### Miscellaneous.

### Salicylate of Soda in Rheumatism.

By A. Clark, M. D.,

Prof. of Pathology and Practical Medicine, College of Physicians and Surgeons, N. Y.

Owing to the interest excited by the recommendation of salicylic acid in rheumatism, the following will be of interest.—(Ed.)

My acquaintance with practice in the New York hospitals began in 1834. Since that time, either at home or abroad, officially or as a volunteer, I have been a very frequent visitor at one or more of these, or similar institutions. In this period of forty-four years the treatment of acute rheumatism has undergone many changes.

At first colchicum and actea racemosa, in tincture, separately or united, were generally relied on; calomel was at the same time often prescribed. After these followed quinine in full doses; then the nitrate of potash, an ounce or more daily, dissolved in a quart or more of water. The free use of lemon-juice was next tried. So far there was reason to doubt whether any of these medicines, except colchicum, aided much in the cure; in other words, whether the disease, after running a certain course, did not terminate spontaneously. The curative effects of colchicum, in full doses, during all these changes were often demonstrated, but serious accidents were occuring in its use, and the profession was searching for a safer treatment.

At length Dr. Fuller, of London, proposed and advocated the alkaline method. The carbonates, or convertible salts of soda and potash, were given in such quantity as to make the urine alkaline in two or three days. Then it really seemed as if we had found a safe and speedy remedy. There were cases that resisted it, but the greater number yielded. The pain subsided on the second, third, or fourth day, the stiffness remaining a day or two longer. This result, so far as it could be obtained, was a great gain. It has been observed that acute inflammations of the pericardium and endocardium which attend acute articular rheumatism, are disposed to wait till the fifth day of the primary disease, or even longer time. To cure the primary disease before the fifth day is, in a great majority of cases, to prevent the cardiac complications. It is true that in rare iustances the cardiac inflammations have been the first manifestation of the rheumatic diathesis, the affection of the joints following one or more days after. It is equally true that the cardiac inflammations have followed that of the joints earlier than the fifth day. Still, the rule has an application sufficiently extended to illustrate one of the important advantages of the Fuller treatment.

Lately the claims of salicylic acid to the first place among the anti-rheumatics have been strongly urged. The following report of its use at Bellevue Hospital may help the profession to form a judgment regarding its virtues, It embraces all the cases received in my wards, from the 1st of April to the 1st of June, that could be called *acute* rheumatism—eleven in all. Short abstracts from the records of these cases have been made at my request by Dr. Kendall and his assistants, from the Hospital Case-Books. Each is full enough to show the general effects of the medicine; but as the effect of it on the temperature and pulse is not always mentioned, I can add that its influence in lowering the fever heat and diminishing the excited pulse were as marked as its power to relieve pain; apparently because it neutralized the rheumatism-poison, and removed the cause of these disturbances. In the the tenth case "*no impression* was made on the disease" for eight days.

The formula used in all these cases is the following:

℞ Acid. salicylic . . . . . . . ʒ iij.

Sodæ bicarbonat . . . . . . ʒ ij.

Glycerine,

Aq . . . . . . . āā ℥ ij.

M.

Of this a tablespoonful was given every two hours for the first day, and afterwards the same quantity six times a day. It is not claimed that this is a perfect salicylate of soda. It was prepared rather rudely by the house-physician, not with reference to atomic neutralizing proportions, but by adding the soda to the suspended acid till he obtained a clear solution. It may be atomically correct or it may not; it matters little, as the combination appears to have curative power. This treatment was begun by Dr. Jacobi, and followed up by me. This report does not include any of Dr. Jacobi's cases, but the house-physician informs me that his results were almost exactly parallel with those here noted. It must be added that in both classes of cases, whenever the joints were very painful, compresses, kept cool by frequent dipping in cold water, were applied to them.

Salicylic acid alone is reported to have cured rheumatism; soda alone cures it; and the formula given here might be considered a union of Fuller's with Stryker's treatment, were it not that the urine has not been noticed to be alkalline, and that the quantity usually taken, even if separated from its acid, is hardly enough to produce alkalinity.

In view of nervous symptoms, more or less alarming, that have been reported as following the use of salicylic acid in some cases; (see Dr. Caro's case, *Med. Record* a few weeks ago); it is important to state that no such results have been noticed at the hospital in those who have taken this salicylate; indeed, no unpleasant affects of any kind have been witnessed, though the quantity of the drug administered is large. I will make no comment on the cases, further than to say that in nine of the eleven the curative action of the medicine was early seen; in two this action was slow and incomplete.

Case 1.—John Clark, æt. 24; single; Ireland; waiter. Admitted April 15, 1876. One previous attack, three years ago. Present began five weeks ago, with pain in soles of feet and some swelling; malaise. Left work three weeks ago. Six days before admission, pain and swelling in knees; since in ankles, hips, elbows, shoulders; right wrist and back affected. On admission knees and ankles moderately swollen, hot and tender, Temp. 102½. Ordered the salicylate as above, with cold compresses to painful joints. April 17th, much improved; some muscular tenderness, and pain in left shoulder only remaining. No more symptoms referable to rheumatism developed, and April 26th, eleven days after admission, *discharged cured.*

This patient had a heart murmur whose connection with present attack is very doubtful.

Case 2.—Chas. Quinn, æt. 22; single; United States; kindling wood. Admitted April 19, 1876. Has had gonorrhœa twice, with rheumatism. Had an acute non-specific attack six years ago, in this Hospital. On admission for present attack, suffering from pain and swelling in left knee and carpo-metacarpal joint of left thumb. Attack began with chill and sweat yesterday. Mitral systolic and aortic direct murmurs. Some pain in præcordia, no friction. Ordered usual treatment. In 24 hours improvement very marked; no paiu. On April 22d, three days after admission, discharged, very much improved. Duration of attack, four days; duration of treatment, three days; result, cure (?).

Case 3.—Wm. H. Welwood; æt. 34; single; United States; horse-dealer. Admitted April 13, 1376. Several previons attacks, subacute in character, with, however, complete recovery. Present attack began eight days ago in right knee, followed soon by pain and swelling in nearly all the joints of body. Was not confined to bed. On admission, left elbow (the only joint much affected) swollen, hot and tender. Temp. 102. Heart normal. Ordered usual treatment. On following day affected joint normal, but stiffness of same joint of opposite side. No further extension or development. On April 17th, four days after admission, discharged cured. Under treatment four days.

Case 4.—H. Heneoer, æt. 30; single; United States; laborer. Admitted April 13, 1876. No history of previous rheumatic attacks. Three days before admission both ankles became swollen, hot and tender. On admission, ankles somewhat swollen and tender. No other joints involved. Temp. 102½. Heart normal. Ordered usual treatment. On following day much better; some tenderness yet. Temperature normal. Treatment continued; improvement rapid, and on April 24th patient discharged cured. In hospital eleven days.

Case 5.—E. Shultze, æt. 28; single; German; bartender. Admitted May 3, 1876. Father had rheumatism; died of cardiac disease. History of seven previous attacks; last in this hospital and in this division.

Present attack began six days ago in ankles; swelling, tenderness, and pain, followed by same in all joints of extremities. On admission, wrists swollen and tender, right elbow very tender, and all joints and limbs stiff. Temp. 103½, pulse 103. Mitral regurgitant murmur. Ordered usual treatment. On following day much better; temp., 101°. May 6, swelling gone; some pain in loins and over heart. May 7, developed symptoms of alcoholism, and was treated accordingly (pot. bromid., etc.). May 10, feels all right, except some soreness in two fingers of left hand. May 16, except stiffness in shoulders, well. May 31, all symptoms absent, except slight stiffness of knees, which disappeared after exercise. Is apparently well. June 3, discharged cured.

Case 6.—Albert Callender, æt. 27; Sweden; laborer. Admitted May 24, 1876. One previous attack, six or seven years ago; present attack began four days ago, after exposure to wet and cold. Began in toes of right foot, swollen and painful; followed by pain and swelling of ankles. Then knees, shoulders, and thumbs became successively involved. Ankles and knees much swollen this morning. Had two profuse sweats before admission. On admission, above condition found. Ordered usual treatment. Cold locally, there being much pain. May 25, patient much better. No pain, except a little in knees. May 27, no pain, no swelling. May 29, discharged cured.

This patient gave a history of dyspnœa on exertion, with frequent spitting of blood, always after exertion. On examination, a mitral obstructive murmur found. Dyspnœa, etc., had existed for four years prior to present attack.

Case 7.—Walter Houseman, æt. 49; single; United States; driver. Admitted May 17, 1876. Brothers and sisters have had "rheumatism." Patent has had numerous attacks like the present. Present attack began one week ago. Shoulder and hand of right side became swollen and tender. On admission, right hand and two middle fingers of same side swollen and red. Little finger much deformed by previous attacks, as also the others, though in less degree. No fever. Ordered usual treatment.

May 20, swelling in hand much less. Very little pain. Beginning to move fingers.

May 20, everything gone but deformity, which existed prior to present attack. He is therefore discharged cured. In hospital thirteen days. There was no evidence of gout other than the residual deformity.

CASE 8.—Timothy Rundell, æt. 26; single; Ireland; laborer. Admitted May 23, 1876. No previous attack. Present attack began three days ago in malaise, etc., followed by stiffness on morning of May 21. Stiffness soon followed by severe pain in both knees and ankles, with considerable fever. Left wrist was soon affected and corresponding elbow. Heart has not troubled him. On admission, above joints were found tender and painful on motion; left wrist and left foot somewhat swollen and red. Ordered usual treatment. Temp. on admission, 103½. May 24, leg same; left wrist better. Back, right wrist, fingers, and elbow getting stiff and sore. No improvement. Heart normal. May 25, considerable improvement. All joints of right arm sore but not swollen. Temp., 102°. May 26, very little pain. Joints weak. May 31, joints of arms stiff since last note. No more pain. Temp. normal. June 2, still some stiffness in shoulders. Otherwise well. Treatment continued. June 5, feels perfectly well; is, therefore, discharged cured. In hospital twelve days.

CASE 9.—Ellen Ryan, æt. 48; married; Ireland; domestic. Admitted May 18, 1876. No history of previous attack. Present attack began three weeks ago in right shoulder. Since then, all joints of extremities have suffered, together with certain muscles. Very little swelling has existed.

On admission, has pain in all joints, but most marked in arms. No swelling of joints except those of right arm, which are swollen and painful. Ordered usual treatment. May 20, much improved. Right arm and left leg (joints) painful only on motion. Affection finally located in shoulders and persisted. Ordered battery as adjuvant to acid. Preferred discharge to use of battery, and was discharged, May 27, improved. Under treatment nine days.

CASE 10.—W. H. Higgins, æt. 20; United states; deck hand. Admitted April 14, 1876. No history of previous attack. Present attack began April 1, in right hip; subsequently all the joints. Lower extremities painful and stiff. Left work to day. On admission, is suffering from intense pain in left ankle, which is swollen, hot, and tender Ordered usual treatment. Improvement in this case was very slow, and no sensible impression made on disease until April 22d, when, with exception of left ankle, symptoms had subsided. Swelling and tenderness persisted here (left ankle), and on May 1, lin. sap. co. and douching ordered. Not much benefit derived from them. Subsequently, tincture of iodine added externally. Slowly improved, and on May 20 was discharged improved.

The acid was continued up to time of discharge.

Under treatment thirty-six days.

CASE 11.—H. G. Phelps, æt. 38; England; printer. Admitted April 17, 1876. Has had repeated attacks of subacute rheumatism.

Twelve days before admission present attack began in right instep, swollen and tender; subsequently "all the joints of body" became involved. Was in bed one week before admission. On admission, all joints somewhat stiff and sore. Ankles and knees slightly swollen. Heart normal. Usual treatment employed. On following day much better. Continued to improve, and on April 24, was discharged cured. In hospital six days.

October 6, 1877. The same treatment has been continued in the first medical division of the hospital, since the date of the last case, above noted, with no change in the results; except, that in two instances, a certain amount of deafness has been produced, like that which follows full doses of quinine, transient, and in the second case, which I examined yesterday, slight in degree.—*Medical Record.*

